# Supplementary material for: Surface enhanced Raman scattering artificial nose for high dimensionality fingerprinting
Source: Nat Commun. 2020 Jan 10;11:207. doi: 10.1038/s41467-019-13615-2 (PMC6954179; doi:10.1038/s41467-019-13615-2)
Supplement: Supplementary file 1 — Supplementary Information [file 41467_2019_13615_MOESM1_ESM.pdf]

Supplementary Information

Surface Enhanced Raman Scattering Artificial Nose for High Dimensionality Fingerprinting

Kim and Thomas *et al*.

Supplementary Discussion 1. Fabrication of SERS-active FASERS substrates

The production of the FASERS Au-nanopillars substrates was achieved *via* colloidal lithography and plasma etching adapted from the reported protocols for silicon nanopillars[1, 2, 3]. Supplementary Figure 1a shows the overall fabrication process comprising four steps: spin coating of polystyrene beads (PS), reactive ion etching (RIE) of the Si_3N_4 coated silicon (Si) wafer substrates for the pillar formation, thermal evaporation of a Cr/Au film, and subsequent SAM functionalization. The change in the surface morphology of the substrates following each step was evaluated with scanning electron microscopy (SEM) images (Supplementary Figure 1b). 300 nm PS beads were used to generate a hexagonal array of beads to act as a mask for RIE. The RIE step enabled etching of the Si_3N_4 layer (~120 nm), which yielded PS capped Si_3N_4 nanopillars (PS-Si_3N_4). It was observed that the RIE step resulted in a roughness change on the surface of the PS beads and their subtle size reduction (~30 nm); both phenomena consistent with reported literature[3]. The subsequent Cr (10 nm) and Au deposition (70 nm) on the PS bead capped Si_3N_4 nanopillars yielded the Au-nanopillars exhibiting gaps of several nanometers between each adjacent dome. It was observed in the SEM images that the Au was successfully deposited on the sidewalls of pillars.

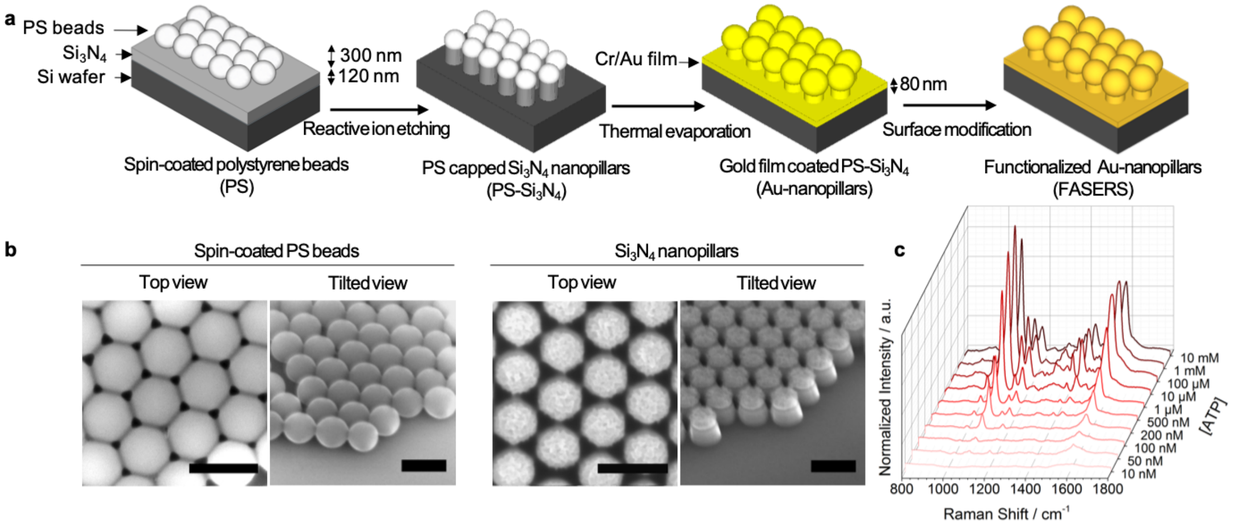

Supplementary Figure 1. Fabrication of FASERS substrates. (a) Schematic illustration of fabrication process of FASERS. (b) Representative SEM images of spin-coated polystyrene beads (PS), and PS-capped Si_3N_4 nanopillars after reactive ion etching (PS-Si_3N_4). SEM images were obtained from top view and angled view from 45° tilted stage without extra metal coating. Scale bar, 400 nm. (c) Series of SERS spectra with varying concentration of 4-aminothiophenol (4-ATP) on non-functionalized Au-nanopillars. Data represent mean of 9 obtained spectra (N = 3, n = 3 spectra) at each concentration.

Supplementary Discussion 2. Characterization of FASERS substrates

2.1. SERS mapping of the Au-nanopillars substrates

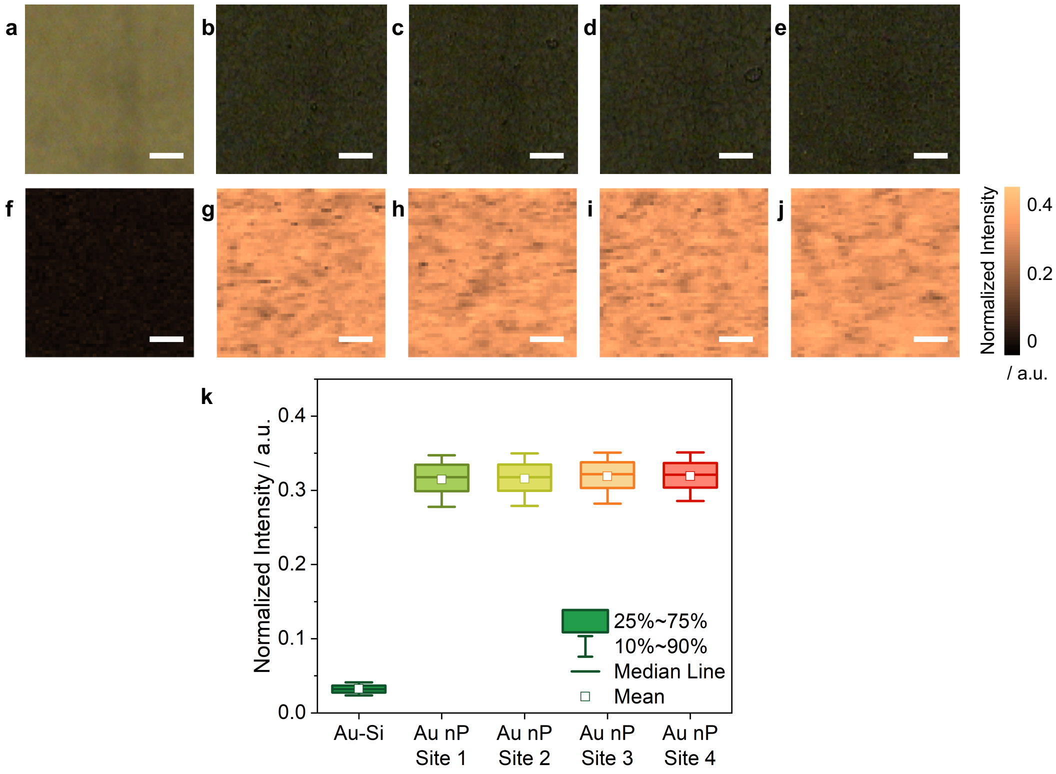

Supplementary Figure 2. SERS mapping of chemisorbed 1 µM 4-mercaptobenzoic acid (4-MBA) on Au-nanopillars and gold film coated Si wafer (Au–Si) substrate. Four randomly-selected spatial locations on the Au-nanopillar substrate (Au nP Site 1–4) were investigated and compared to one location on a Au–Si substrate for control. (a–e) Optical microscope image of (a) Au–Si, (b) Au nP Site 1, (c) Au nP Site 2, (d) Au nP Site 3, (e) Au nP Site 4. (f–j) SERS mapping (50 µm x 50 µm, pixel size of 1 µm^2, peak at 1074.7 cm^{-1}) of corresponding location: (f) Au–Si, (g) Au nP Site 1, (h) Au nP Site 2, (i) Au nP Site 3, (j) Au nP Site 4. Scale bar represents 10 µm. Each spectrum was smoothed, baseline subtracted and normalized by the area under the curve. The maximum value of the intensity bar corresponds to the maximum intensity out of all the measurements performed on all of the different sites. (k) Normalized SERS intensity of peak at each pixel (50 x 50) of the scan. Significant SERS enhancement of 4-MBA signals across the entire scanned area were observed compared to those of Flat Au surface (Au–Si). Importantly, we have observed no/little apparent mean signal variation between the different spatial location, indicating a good spatial reproducibility of the enhancement.

2.2. Wettability of FASERS substrates

The wettability of each functionalized substrate was influenced by the head group and chain length of each SAM ligand molecule. Hydrophilic headgroups (hydroxyl-, carboxyl- and amine-terminated SAMs) resulted in a reduced contact angle with increasing chain length. This is likely attributed to the longer chain SAMs having formed more ordered structures than the shorter molecules due to the stronger van der Waals interactions between longer adjacent alkyl chains[4]. The nanopillar structures resulted in a slightly increased (~5°) contact angle compared to that of flat Au–Si (Supplementary Figure 3b–d). Compared to Au–Si, all the SAMs with hydrophilic end-groups (hydroxyl, carboxyl, amine) exhibited increased hydrophilicity while the hydrophobic end-groups (alkyl) showed increased contact angle, consistent with the Wenzel's theory and Cassie and Baxter theory, respectively[5, 6, 7].

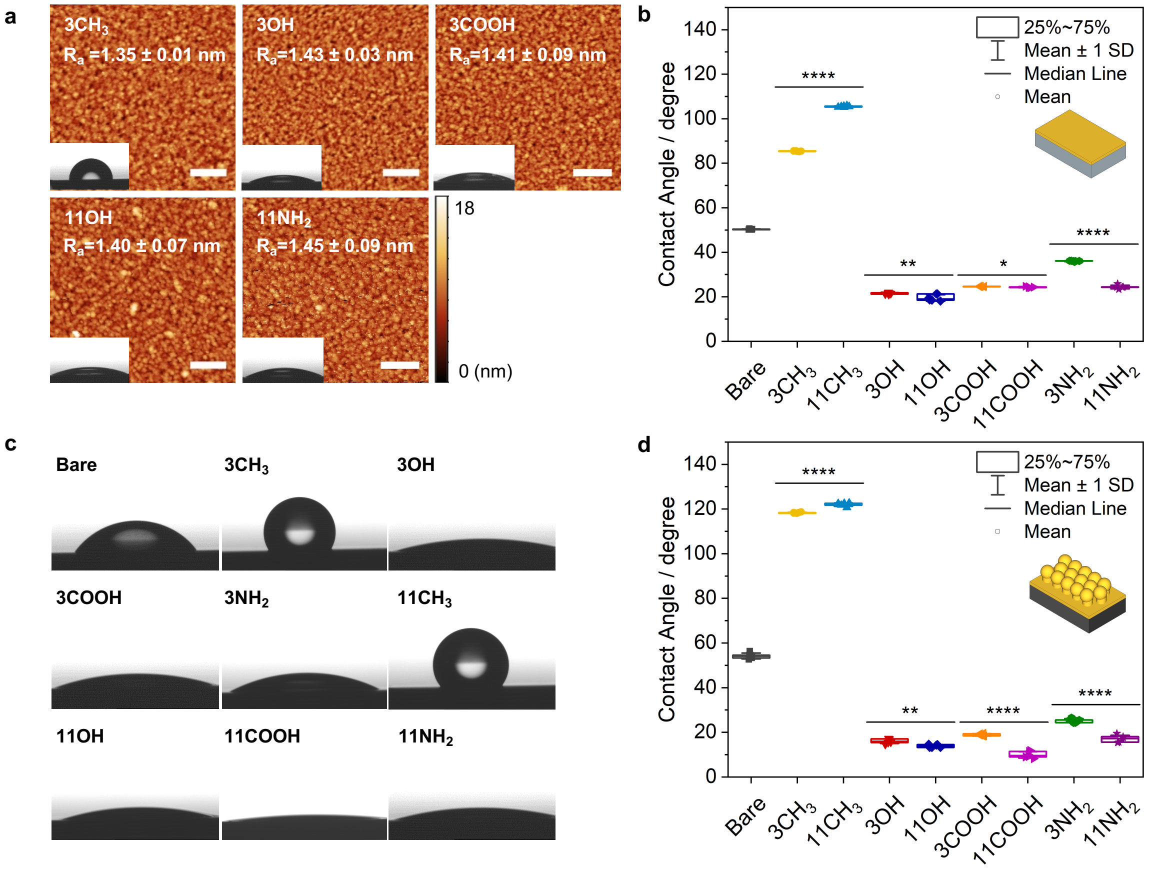

Supplementary Figure 3. Wettability and surface roughness of functionalized gold film coated Si wafer (Au–Si) and Au-nanopillars. (a) AFM images of functionalized Au–Si with various SAM molecules (from top-left to bottom-right): 1-propanethiol ($3CH_3$) 3-mercapto-1-propanol (3OH), 1-undecanethiol ($11CH_3$), 3-mercaptopropionic acid (3COOH), 11-mercapto-1-undecanol (11OH), 11-amino-1-undecanethiol ($11NH_2$). Scale bar, 400 nm. Average of the mean roughness (R_a) of each surface was noted in the image with ± 1 s.d. (n = 3 scans). Inset images indicate the range of water contact angles observed at each surface. (b) Water contact angle measurements of functionalized Au–Si. Data represent the mean + 1 s.d. angles, measured from 3 droplet images at right and left side (N = 3, n = 2). ****$p < 0.0001$, ***$p < 0.001$, **$p < 0.01$ and *$p < 0.05$ based on one-way ANOVA and Tukey's honest significance test. (c) Representative images of water contact angle on the functionalized Au-nanopillars: non-functionalized (bare), 1-propanethiol ($3CH_3$), 3-mercapto-1-propanol (3OH), 3-mercaptopropionic acid (3COOH), 3-amino-1-propanethiol ($3NH_2$), 1-undecanethiol ($11CH_3$), 11-mercapto-1-undecanol (11OH), 11-mercaptoundecanoic acid (11COOH), 11-amino-1-undecanethiol ($11NH_2$). (d) Water contact angle measurements of functionalized Au-nanopillars. Data represents contact angles measured from 3 droplet images at both sides (N = 3, n = 2). ****$p < 0.0001$, ***$p < 0.001$, **$p < 0.01$ and *$p < 0.05$ based on one-way ANOVA and Tukey's honest significance test.

2.3. Tentative peak assignment of SERS spectra from self-assembled monolayers

The C–C stretching region (1000–1500 cm^{-1}) of the monolayers contains information about the conformational behavior of the alkyl chain, arising from the *gauche* and *trans* conformers of C–C bonds.

The prominent peaks around 1100–1130 cm^{-1} correspond to (C–C) *trans*-conformers, the positions of which agree well with a number of previous studies[8, 9, 10, 11, 12]. Regarding the relatively strong intensity of (C–C) stretching of 1-propanethiol and 1-undecanethiol compared to other short thiolates, this is attributed to the 1-alkanethiol molecules having the lowest symmetry amongst the C_s point group, therefore the vibrations of the adsorbed molecule exhibit a component perpendicular to the surface in all orientations[9, 11]. As the vibration components along the z-axis (i.e. zz tensor component) are enhanced to a larger extent than those with xz or yz components, the C–C components of 1-alkanethiol show a large contribution of stretching vibration of *trans*-conformers[9, 11]. On the other hand, the short-chain alkanethiol with a substituted terminal group can also interact with the metal surface[10, 13, 14, 15, 16, 17]. In particular, the terminal carboxylic and amino group are known to exhibit a relatively higher concentration of *gauche* conformers via double bonding to the metal surface[10, 13, 14, 15], which leads to minimized stretching vibration of *trans*-conformers when compared to 1-propanethiol.

For monolayers with 11 carbon atoms (11CH_3, 11OH, 11COOH, 11NH_2), the peak corresponding to stretching vibration of the C–C *trans*-conformer (i.e. $v_s(C–C)_T$) are the most clearly observable of the signals. This is consistent with the findings of previous studies in which monolayer structures begin to resemble that of the solid from carbon number 8[12], and the symmetric stretching vibrations of C–C bonds are the largest contribution from the tensor component along the axis of the vibration[11].

Supplementary Table 1. Tentative assignments of prominent peaks of SAM monolayers. Each SAM is referred to as: 1-propanethiol (3CH_3), 3-mercapto-1-propanol (3OH), 3-mercaptopropionic acid (3COOH), 3-amino-1-propanethiol (3NH_2), 1-undecanethiol (11CH_3), 11-mercapto-1-undecanol (11OH), 11-mercaptoundecanoic acid (11COOH), 11-amino-1-undecanethiol (11NH_2).

SAM-forming molecules	3CH_3	3OH	3COOH	3NH_2	11CH_3	11OH	11COH	11NH_2
				Raman shift (cm^{-1})				
[a,b] CH_2 $(rock)_G$ **[11, 12]**	838				860.0			
[a,b] $CH_3$$(rock)_T$ **[11, 12]**	889<br>1002				891<br>1004	999	1003	1004
v($C–COO^-$) **[16, 17]**			921					
[a] v$(C–C)_T$ **[11, 12]**	1028	1028						
[a] v$(C–C)_G$ **[11]**<br>[a,c] $v_s(C–C)_T$ **[9, 12]**			1042		1065<br>1074	1062	1062	1096
[a,c] $v_s(C–C)_T$ **[9, 11, 12]**	1087			1119	1125	1108	1099	1107
[b] CH_2 wag **[11, 12]**	1283.8	1248		1281	1297	1298	1272<br>1301	1258
[b,d] CH_3 sy.def **[12]**	1324<br>1384<br>1415	1384	1384<br>1415	1384<br>1437	1323<br>1344<br>1384<br>1437	1324<br>1345<br>1358<br>1383<br>1397	1322<br>1345<br>1362<br>1384<br>1396	1304<br>1321<br>1344<br>1383
[b,d] CH_3 as.def **[12]**	1454		1446	1448	1449	1437	1447	1442

[a] $( )_G$: *gauche*-conformer, $( )_T$ *trans*-conformer. [b] def.: deformation, wag.: wagging, rock: rocking. [c] v_s: stretching. [d] sy.: Symmetric, as.: asymmetric.

2.4. XPS analysis

In all 8 SAM-functionalized surfaces, clear S *2p* peaks were identified and assigned to bound-thiolate (Au–S) which is reported to be located in a ~1.5 eV lower binding energy region than that of unbound thiol (S–H)[18]. It was observed that the short thiolates were bound with no detectable contaminants, while most of the long thiolates showed a signal correlating to small fraction of non-bound thiolates, which is commonly detected for ethanolic immersion methods[4, 18]. In particular, the C *1s* peak of the alkyl-terminating SAM (Supplementary Figure 4) was composed of C–C bonding while the hydroxyl-terminating SAM exhibited an additional peak arising from C–O bonds (Figure 2h). The carboxyl end-group was identified in emerging peaks related to C–O, C=O, C–OH, and O–C=O bonds, while the peak corresponding to the amine end-group was fitted to C–C and C–N bonds with C–O possibly from surface-bound water[19]. The bare Au surface C *1s* peak showed a small amount of non-volatile hydrocarbon contaminants (C–C bond), which are commonly detected due to the high surface energy of gold[20, 21].

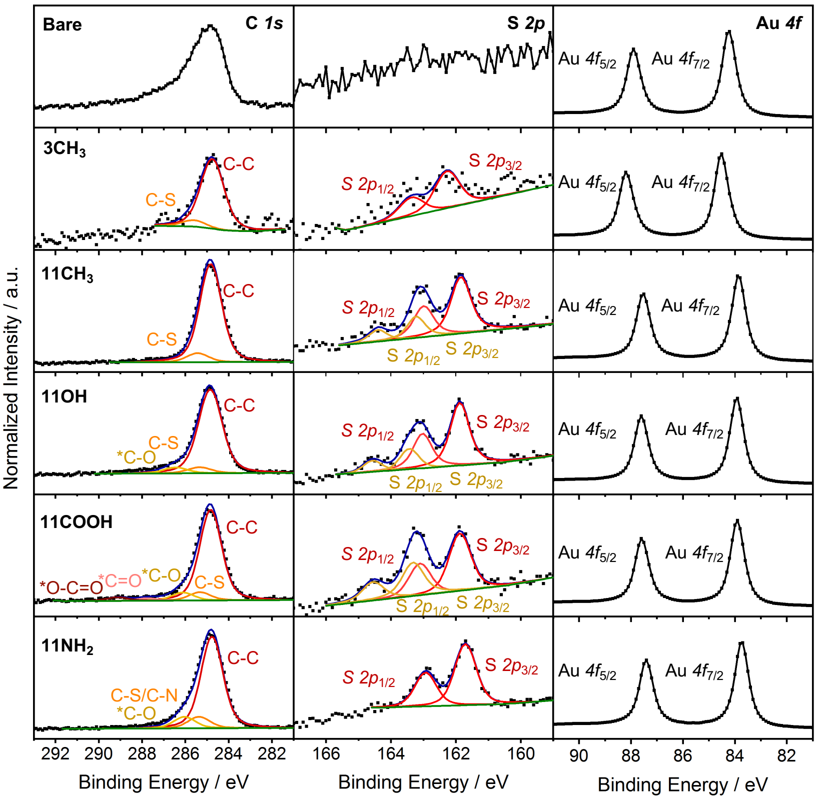

Supplementary Figure 4. High-resolution XPS spectra of C *1s*, S *2p* and Au *4f* for non-functionalized and SAM-functionalized Au–Si. Each SAM is referred to as: non-functionalized (bare), 1-undecanethiol (11CH$_3$), 11-mercapto-1-undecanol (11OH), 11-mercaptoundecanoic acid (11COOH), 11-amino-1-undecanethiol (11NH$_2$). * Refers to the weak peaks that are expected from the chemical structure of the molecules used. The peak shifting in the 3-carbon SAMs is likely attributed to attenuation of X-ray intensity due to differing degrees of overlayering on top of the SAM.

Supplementary Discussion 3. Tentative peak assignment of SERS spectra from model analytes

The prominent SERS bands of *p*-PDA were located at 1169 cm^{-1}, 1496 cm^{-1}, 1601 cm^{-1} with a shoulder at 1627 cm^{-1}, which are consistent with the previously reported SERS spectrum of *p*-PDA on gold nanoparticles[22]. Each band can be assigned to C–N stretching/in-plane NH_2 wagging, in-plane C–N stretching, and in-plane NH_2 scissoring, respectively. The spectra of 4-APA have two prominent bands at 1175 cm^{-1} and 1601 cm^{-1}, which can be tentatively assigned to C–N stretching[22] and the ring breathing mode of the aromatic ring,[23] respectively. Figure 3d and 3e showed resultant signatures of R6G and FA from FASERS. The peak positions of R6G are in accordance with previously reported spectra, obtained both experimentally[24, 25, 26] and computationally[27, 28]. Several prominent bands include 1186 cm^{-1}, 1312 cm^{-1}, 1602 cm^{-1}, and 1646 cm^{-1}, which were tentatively assigned to in-plane ring deformation, xanthene ring breathing, asymmetric C=C stretching mode and xanthene ring stretching, respectively. The characteristic bands of FA were also in good agreement with previously reported SERS spectra of FA adsorbed on silver and gold substrates[29, 30]. The SERS bands correspond to NH_2 scissoring/C–O stretching (1625 cm^{-1}), aromatic ring breathing (1595 cm^{-1}), in-plane N–H vibration (1563 cm^{-1}), CH_2 wagging/in-plane C–H deformation (1321 cm^{-1}), and CH_2 aliphatic twist (1181 cm^{-1}), respectively[31]. Further details regarding peak assignments for these molecules are summarized in Supplementary Table 2–3.

Supplementary Table 2. Summary of tentative peak assignment of SERS peaks of 4-aminophenylacetic acid (4-APA) and *p*-phenylenediamine (*p*-PDA).

4-Aminophenylacetic acid (4-APA)		*p*-Phenylenediamine (*p*-PDA)	
Peak position	Vibration mode	Peak position	Vibration mode
1601 cm^{-1}	Aromatic ring breathing[23]	1627 cm^{-1}	In-plane NH_2 scissoring[22]
1175 cm^{-1}	C–N stretching /In-plane NH_2 wagging[22]	1601 cm^{-1}	Aromatic ring stretching /In-plane NH_2 scissoring[22]
1075 cm^{-1}	Aromatic ring breathing[23]	1496 cm^{-1}	In-plane C–N stretching/ C–C stretching[22]
		1230 cm^{-1}	C–N stretching/ In-plane C–C bending[22]
		1169 cm^{-1}	C–N stretching/ In-plane NH_2 wagging[22]

Supplementary Table 3. Summary of tentative peak assignment of SERS peaks of folic acid (FA) and rhodamine 6G (R6G).

Folic acid (FA)		Rhodamine 6G (R6G)	
Peak position	Vibration mode	Peak position	Vibration mode
1625 cm^{-1}	NH_2 scissoring/C=O stretching[31]	1646 cm^{-1}	Xanthene ring stretching[24, 28] /In-plane C–H bending[28]
1595 cm^{-1}	Aromatic ring breathing[31]	1602 cm^{-1}	Asymmetric C=C stretching[27]
1563 cm^{-1}	Aromatic ring stretching /In plane N–H vibration[31]	1509 cm^{-1}	Xanthene ring stretching[24, 28] /C–N stretching/C–H bending /N–H bending[28]
1495 cm^{-1}	CH_2 scissoring /In-plane C–H deformation[31]	1362 cm^{-1}	Xanthene ring stretching[24, 28] /in-plane C–H bending[28]
1321 cm^{-1}	CH_2 wagging /Aromatic C–H stretching[31]	1312 cm^{-1}	Xanthene ring breathing /N–H bending/CH_2 wagging[28]
1181 cm^{-1}	In-plane C–H bending /CH_2 (aliphatic) twist[31]	1186 cm^{-1}	In-plane ring deformation /C–H bending/N–H bending[28]
965 cm^{-1}	O–H bending, CH_2 twist/rock[31]		

Supplementary Discussion 4. Molecular dynamics simulation: computational details

All-atom molecular dynamics (MD) simulations were performed to structurally characterize the eight SAM-functionalized surfaces of differing composition and explore the mechanisms of binding of the two small molecules, *p*-PDA and 4-APA, with the SAMs and bare gold. Simulations consisted of two stages: (i) monolayers were thermally equilibrated to characterize their interfacial structure and properties, (ii) the equilibrium SAM structures were then used to explore interactions with individual analyte molecules.

Each system contained a close-packed face-centered cubic Au(111) slab of periodic unit cell dimensions 4.1 × 4.4 nm^2 in the lateral (X-Y) directions and 8 layers (1.7 nm) of fixed gold atoms in the Z direction (surface normal). On each exposed gold slab surface, 80 monolayer molecules were initially placed upright with their sulfur head groups arranged in a hexagonal (√3 ×√3)R30° structure[32] relative to the underlying Au(111) lattice (Supplementary Figure 5). This produced a packing density of 22.33 $Å^2$ / chain, which corresponds to a fully saturated coverage and maximum packing density[32]. A 6 nm spacer was introduced between the two SAMs in each simulation cell (Supplementary Figure 5a–b) ensuring that the two surfaces (top/bottom) were sufficiently apart to prevent self-interactions and allowing for property statistics to be gathered from two surfaces per simulation while minimizing computational expense. The SAMs modelled are commensurate with the 8 different SAM-functionalized Au-nanopillar substrates, which differ in terminal group chemistry and chain length, including: 1-propanethiol ($3CH_3$), 3-mercapto-1-propanol (3OH), 3-mercaptopropionic acid (3COOH), 3-amino-1-propanethiol ($3NH_2$), 1-undecanethiol ($11CH_3$), 11-mercapto-1-undecanol (11OH), 11-mercaptoundecanoic acid (11COOH), and 11-amino-1-undecanethiol ($11NH_2$). The analytes were modelled in the same buffer conditions as per experiment, where *p*-PDA (pH 5) had one of the amine groups protonated and 4-APA (pH 7.5) had the carboxyl group deprotonated. The different pH conditions for each analyte were also reflected in the SAM composition. Carboxyl terminated SAMs 3COOH and 11COOH have surface pK_a values of 5.2[33] and 5.0[34] respectively, thus SAMs composed of these ligands were modelled with two different protonation states: all carboxyl groups deprotonated (COO^-, pH 7.5); and a 1:1 alternating mixed state of neutral (COOH) and deprotonated (COO^-) carboxyl groups (pH 5, Supplementary Figure 5b). Amine terminated SAMs $3NH_2$ and $11NH_2$ have surface pK_a values of 8.5[35] and 8.9[34] respectively, and therefore were modelled with all molecules in their protonated state (NH_3^+, pH 5 and 7.5). Systems were solvated with explicit water (density of approx. 1 g/cm^3), counter ions (to ensure charge neutrality) and in the second stage of simulations, two *p*-PDA or 4-APA molecules were also added.

Computations were performed using the GROMACS 4.6.5 software package[36] in conjunction with the GolP-CHARMM[37] force field and the modified TIP3P[38] water model. The GolP-CHARMM force field was employed since it was specifically designed to capture organic species adsorption at aqueous Au(111) interfaces via a combination of experimental and first-principles data, and the force field contains explicit terms to describe the dynamic polarization of Au atoms, chemisorbing species, and interactions between sp^2 hybridized carbon atoms and Au. To emulate the strong Au–S bonding formed between thiols and gold,[39] sulfur atoms were position restrained to the X–Y plane ~2.3 Å[40, 41] above the Au(111) surface. Together with a Lennard-Jones Au–S non-bonded potential (σ = 2.05 Å, ε = 3.2 kJ/mol), this allowed for the lateral mobility and reorienting of SAM molecules on the Au surface.[42] The ParamChem server (https://paramchem.org) was used to obtain CHARMM compatible parameters[43] and atomic partial charges for the *p*-PDA, 4-APA and SAM molecules.

Non-bonded long-range electrostatic interactions were evaluated using the Particle Mesh Ewald (PME) method with a real space cut-off of 12 Å and a 1.2 Å fast Fourier transform (FFT) grid spacing.

Van der Waals interactions were computed with a force-switch cut-off starting at 0.9 Å and ending at 10 Å. Energy minimization (EM) was performed using the steepest descent algorithm to remove steric clashes. The MD was performed in the canonical (NVT) ensemble with temperature maintained at 300 K using the Nosé-Hoover thermostat[44, 45], an integration time step of 1 fs, and the LINCS algorithm[46] to constrain bonds with hydrogen atoms. Analysis trajectories were outputted at a rate of one frame every 2 ps.

The simulation protocol for stage (i) and (ii) of the simulations was as follows. In stage (i), EM was followed by a series of 200 ps long MD simulations that sequentially increased the number of water molecules to fill the cavities formed by the relaxing SAM, until the solvent density plateaued at 1 g/cm^3 in the center of the unit cell. Subsequently, MD of each system was performed for 100 ns to determine the equilibrium structure and properties for each SAM. This process was independently repeated three times using different initial atomic velocities effectively resulting in 600 ns of statistics per SAM system (100 ns × 2 surfaces × 3 repeats). Analysis was conducted on the equilibrated part of the trajectory of each simulation (final 50 ns), determined by monitoring SAM molecule RMSD and average tilt angle convergence (not shown). The final frame from each simulation in stage (i) was then extracted (including the solvent) and used as the initial structure for the stage (ii) simulations. In stage (ii), two identical but randomly oriented *p*-PDA or 4-APA molecules were added to each unit cell at positions ~2 nm from each SAM–solvent interface (top/bottom) and over 3 nm apart from each other to ensure there were no immediate self-interactions and close contacts with each SAM. Any water molecules within a radius of 1.2 Å of the analytes were removed, EM was conducted, and 10 ns of NVT MD was performed. This was repeated five times per SAM configuration with random initial analyte orientations to increase statistics. A total of 300 ns per system (10 ns x 2 analytes x 5 orientations x 3 initial SAM configurations) was collected for data analysis. To investigate the interactions of *p*-PDA and 4-APA with gold specifically, MD simulations were also performed of individual analyte in the presence of a bare Au(111) surface, using the same approach as described above. Overall, stage (ii) resulted in a total of 5.4 μs of simulation time.

Model building, statistical analysis, and visualization of the data was performed using the GROMACS 4.6.5 suite analysis tools[36] and the VMD 1.9.3 package.[47] Supplementary Figure 6 schematically depicts the main features used to characterize the MD obtained atomistic assemblies. Monolayer thickness (x) and tilt angle (θ) were used to describe the equilibrium SAM structures (Supplementary Table 4), while the configuration of 4-APA and *p*-PDA at each SAM (Figure 4, Supplementary Figure 7, Supplementary Table 5–6) were characterized with four parameters: analyte–Au distance (d), analyte orientation angle (φ), benzene orientation angle (ψ), and the percentage of simulation time the analyte spent in close proximity (< 0.6 nm) to the SAM/Au surface. Monolayer thickness, x, was measured as the average perpendicular separation between the gold surface atoms and SAM molecule terminal heavy atoms, i.e. methyl carbon (CH_3), hydroxyl oxygen (OH), carboxyl(ate) oxygens (COOH/COO^-), and amine nitrogen (NH_3^+). SAM tilt angle, θ, was determined as the angle of the molecular backbone (principal axis of inertia) with respect to the substrate normal. 11-carbon SAM molecules with a tilt angle > 45° were considered as collapsed chains/defects present on the SAM surfaces, and therefore to avoid skewed data and obtain accurate representative estimates for x and θ, measurements from these chains were excluded from the average values reported in Supplementary Table 4. Analyte–Au distance, d, was measured as the distance between analyte center-of-mass and the nearest Au(111) surface atom. The analyte molecular orientation relative to Au(111) surface, φ, was measured as the angle between the analyte molecular vector and the gold substrate normal vector. For *p*-PDA, the molecular vector was defined between nitrogen on NH_2 and nitrogen on NH_3^+, while for APA this was between nitrogen on the NH_2

and carbon on COO^-. The normal vector of the analyte benzene ring and the normal vector of the gold surface were used to measure the angle *ψ*. Average values presented in Supplementary Table 5–6 are only for simulation frames where analytes were proximate (< 0.6 nm) from the SAM/Au interface.

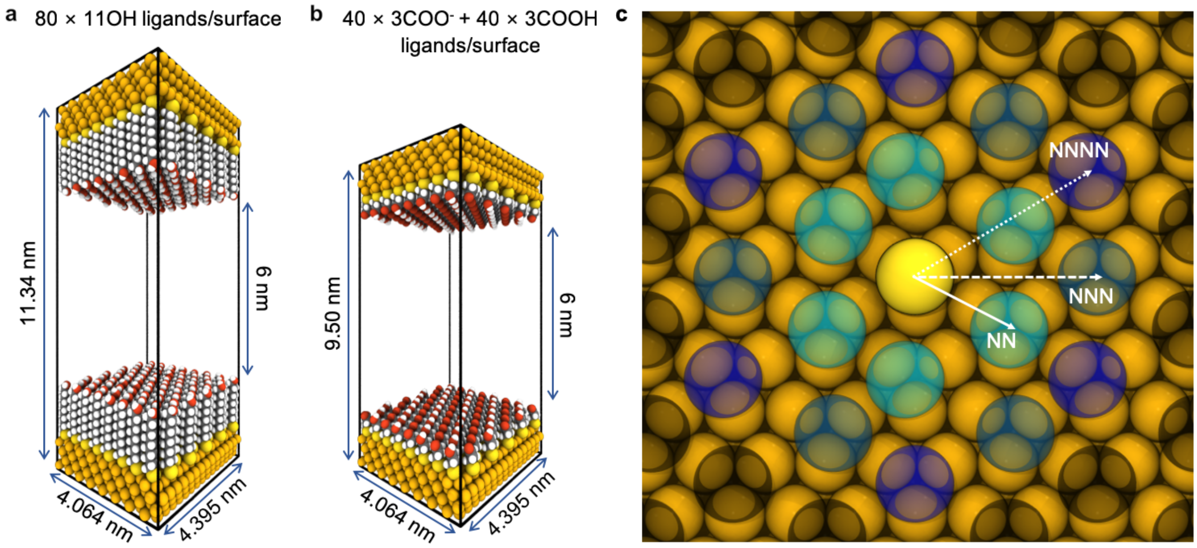

Supplementary Figure 5. Molecular dynamics simulation models. Representative examples showing initial periodic simulation cell for SAMs (a) 11-mercapto-1-undecanol and (b) 3-mercaptopropionic acid (at pH 7.5). Atoms are colored as follows: Au – gold, S – yellow, C – green, H – white, O – red. Water molecules are hidden for clarity. (c) Hexagonal arrangement of sulfur atoms on the Au(111) surface, which produces a ligand packing density of 22.3 Å^2/chain (or 4.48 chains/nm^2). For a given sulfur atom (yellow), arrows point to the nearest neighbor (NN), next nearest neighbor (NNN), and next, next nearest neighbor (NNNN) sulfur atoms, which are shown in gradated shades of blue and are 5.07 Å, 8.79 Å, and 10.15 Å away from the original sulfur atom, respectively.

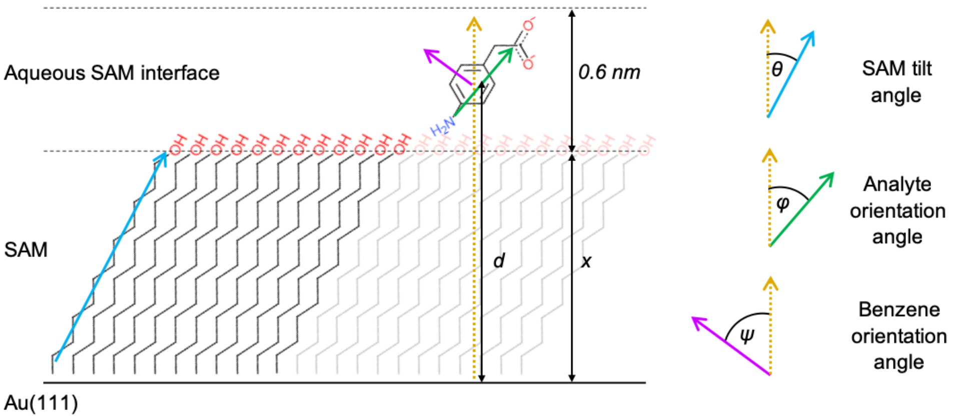

Supplementary Figure 6. Structural parameters used to characterize SAM-functionalized surfaces and analyte-SAM properties obtained by MD simulations. Vectors: SAM molecular backbone vector (blue), normal vector to Au(111) (yellow), analyte molecular vector (green) and normal vector to analyte benzene ring (purple). Angles: SAM tilt angle (*θ*), analyte orientation angle (*φ*), benzene orientation angle (*ψ*). Distances: Monolayer thickness (*x*), Analyte center-of-mass to Au(111) (*d*), analyte–SAM contact proximity (0.6 nm).

Supplementary Table 4. Average monolayer properties determined from molecular dynamics (MD) simulations. Each SAM is referred to as: non-functionalized (bare), 1-propanethiol (3CH$_3$), 3-mercapto-1-propanol (3OH), 3-mercaptopropionic acid (3COOH), 3-amino-1-propanethiol (3NH$_2$), 1-undecanethiol (11CH$_3$), 11-mercapto-1-undecanol (11OH), 11-mercaptoundecanoic acid (11COOH), 11-amino-1-undecanethiol (11NH$_2$).

SAM molecule	Thickness, x (nm) [a]	Tilt angle, θ (deg) [b]	Collapsed chains [c]
11NH$_2$	1.64 ± 0.17*	12.5 ± 8.8*	6.8% ± 1.5%
11COOH pH 5	1.49 ± 0.11*	22.1 ± 5.8*	1.1% ± 1.6%
11COOH, pH 7.5	1.54 ± 0.15*	16.6 ± 9.0*	4.5% ± 2.0%
11OH	1.50 ± 0.04*	32.7 ± 2.5*	0.3% ± 0.9%
11CH$_3$	1.42 ± 0.05*	31.6 ± 3.0*	0.7% ± 0.6%
3NH$_2$	0.72 ± 0.05	18.5 ± 10.7	-
3COOH, pH 5	0.61 ± 0.08	26.0 ± 14.5	-
3COOH, pH 7.5	0.66 ± 0.06	22.4 ± 11.4	-
3OH	0.62 ± 0.09	29.2 ± 17.0	-
3CH$_3$	0.57 ± 0.05	24.6 ± 12.5	-

[a]Monolayer thickness measured as average distance from Au(111) surface to the terminal heavy atoms of the SAM molecules. [b]Angle measured between the normal to the Au(111) surface and the principal axis (with the smallest moment of inertia) of each SAM molecule. [c]11-carbon SAM chains with a measured tilt > 45° are considered defects (max. of 6.8% collapsed chains per frame). *Measured excluding collapsed chains. Packing density = 22.33 Å^2/chain = 4.48 chain/nm^2.

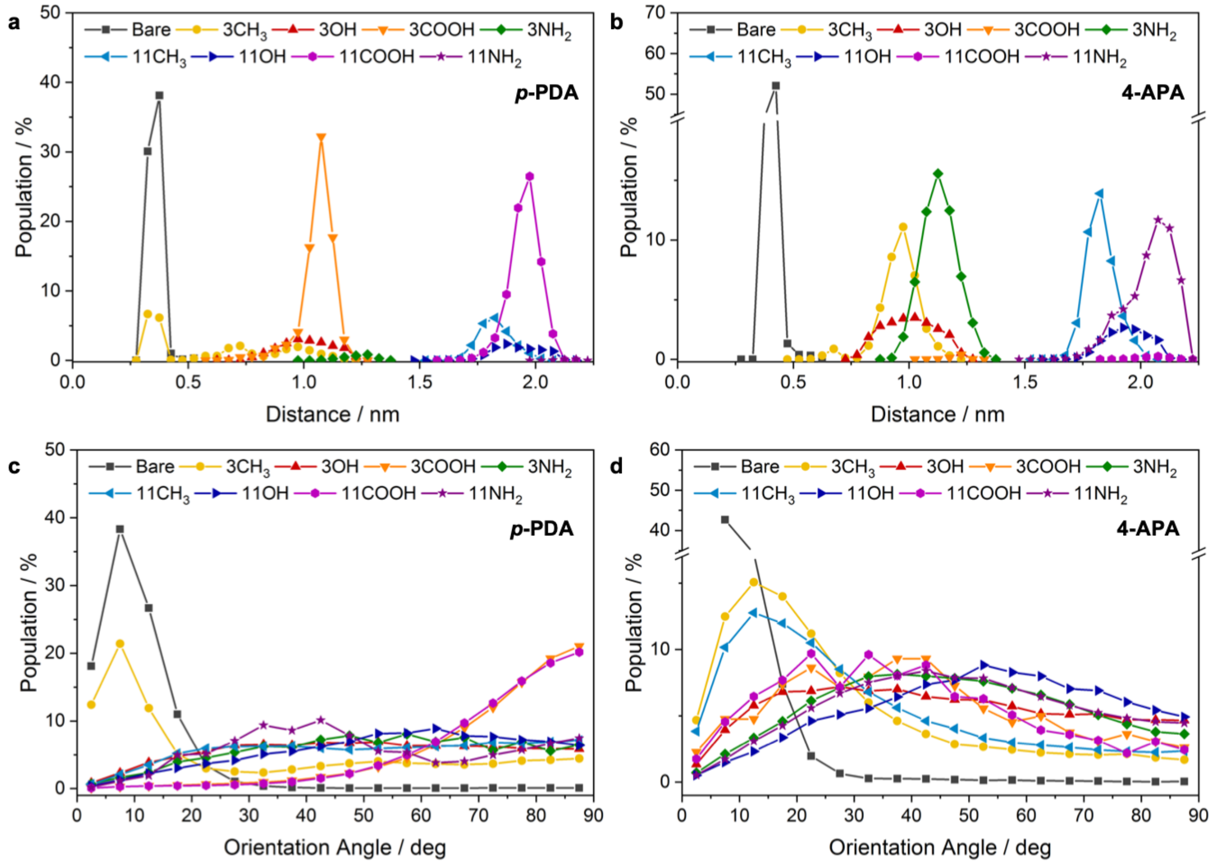

Supplementary Figure 7. Distribution of molecular properties of *p*-phenylenediamine (*p*-PDA) and 4-aminophenyacetic acid (4-APA) on SAM-functionalized surface by MD simulations. Perpendicular distances of (a) *p*-PDA and (b) 4-APA to Au(111). Analyte centers-of-mass are used to measure the distance to the closest Au surface atom and populations are calculated from each system's ensemble of simulation snapshots. For clarity, only populations for analytes in close proximity (< 0.6 nm) to a SAM/Au interface are shown. Benzene orientation angles of (c) *p*-PDA and (d) 4-APA relative to the Au surface for analytes close (< 0.6 nm) to SAM/Au. Angles of 0° and 90° indicate when the benzene ring is parallel and perpendicular to the underlying gold surface, respectively.

Supplementary Table 5. Summary of molecular interactions of *p*-phenylenediamine (*p*-PDA, pH 5) on different SAM-functionalized surfaces. Each SAM is referred to as: non-functionalized (bare), 1-propanethiol ($3CH_3$), 3-mercapto-1-propanol (3OH), 3-mercaptopropionic acid (3COOH), 3-amino-1-propanethiol ($3NH_2$), 1-undecanethiol ($11CH_3$), 11-mercapto-1-undecanol (11OH), 11-mercaptoundecanoic acid (11COOH), 11-amino-1-undecanethiol ($11NH_2$).

SAM molecule	Interaction time with SAM/Au [a, b]	Distance to Au(111), *d* (nm) [a, c]	*p*-PDA orientation angle, φ (deg) [a, d]	Benzene orientation angle, ψ (deg) [a, e]	Closest distance, *d* (nm) (φ, ψ (deg))
Bare Au(111)	69.84%	0.36 ± 0.02	83 ± 7	10 ± 8	0.30 (82, 11)
$11NH_2$	0.35%	2.14 ± 0.03	60 ± 26	50 ± 22	2.05 (61, 28)
11COOH	80.77%	1.95 ± 0.06	155 ± 18	73 ± 15	1.65 (90, 40)
11OH	11.87%	1.92 ± 0.10	64 ± 31	55 ± 21	1.52 (72, 38)
$11CH_3$	21.77%	1.83 ± 0.07	68 ± 16	51 ± 24	1.58 (40, 52)
$3NH_2$	2.64%	1.23 ± 0.06	88 ± 32	52 ± 22	1.01 (90, 22)
3COOH	79.19%	1.06 ± 0.07	153 ± 21	73 ± 15	0.63 (115, 43)
3OH	18.84%	1.01 ± 0.11	73 ± 29	50 ± 23	0.68 (105, 20)
$3CH_3$	27.58%	0.62 ± 0.28	91 ± 33	33 ± 29	0.31 (86, 5)

[a]When in close contact (< 0.6 nm) with Au/SAM. [b]Percentage of frames where the analyte is in contact with Au/SAM. [c]Measured as the analyte center-of-mass to the closest Au surface atom. [d]Relative to Au(111): angles < 90° indicate that the neutral amine (NH_2) is pointing towards the SAM/Au surface, and > 90° represent the charged amine (NH_3^+) is facing the SAM/Au surface. [e]Relative to Au(111): angles range from 0° to 90°, which denote when the benzene ring is parallel and perpendicular to the underlying gold surface.

Supplementary Table 6. Summary of molecular interactions of 4-aminophenylacetic acid (4-APA, pH 7.5) on different SAM-functionalized surfaces. Each SAM is referred to as: non-functionalized (bare), 1-propanethiol ($3CH_3$), 3-mercapto-1-propanol (3OH), 3-mercaptopropionic acid (3COOH), 3-amino-1-propanethiol ($3NH_2$), 1-undecanethiol ($11CH_3$), 11-mercapto-1-undecanol (11OH), 11-mercaptoundecanoic acid (11COOH), 11-amino-1-undecanethiol ($11NH_2$).

SAM molecule	Interaction time with SAM/Au [a, b]	Distance to Au(111), *d* (nm) [a, c]	*p*-PDA orientation angle, φ (deg) [a, d]	Benzene orientation angle, ψ (deg) [a, e]	Closest distance, *d* (nm) (φ, ψ (deg))
Bare Au(111)	74.91%	0.41 ± 0.02	74 ± 5	9 ± 7	0.34 (86, 5)
$11NH_2$	54.11%	2.04 ± 0.10	124 ± 30	49 ± 21	1.55 (88, 13)
11COOH	0.76%	2.05 ± 0.06	103 ± 25	39 ± 21	1.88 (128, 59)
11OH	13.57%	1.94 ± 0.09	70 ± 32	52 ± 21	1.62 (75, 25)
$11CH_3$	41.79%	1.83 ± 0.06	78 ± 15	32 ± 22	1.58 (47, 39)
$3NH_2$	59.53%	1.13 ± 0.07	129 ± 19	47 ± 21	0.91 (125, 15)
3COOH	0.59%	1.21 ± 0.03	100 ± 26	41 ± 22	1.09 (83, 35)
3OH	23.37%	1.00 ± 0.11	80 ± 27	45 ± 24	0.73 (77, 68)
$3CH_3$	37.63%	0.96 ± 0.09	76 ± 18	29 ± 22	0.53 (52, 48)

[a]When in close contact (< 0.6 nm) with Au/SAM. [b]Percentage of frames where the analyte is in contact with Au/SAM. [c]Measured as the analyte center-of-mass to the closest Au surface atom. [d]Relative to Au(111): angles < 90° indicate that the amine (NH_2) is pointing towards the SAM/Au surface, and > 90° represent the carboxyl (COO^-) is facing the SAM/Au surface. [e]Relative to Au(111): angles range from 0° to 90°, which denote when the benzene ring is parallel and perpendicular to the underlying gold surface.

Supplementary Discussion 5. Multivariate Analysis on Biological Samples

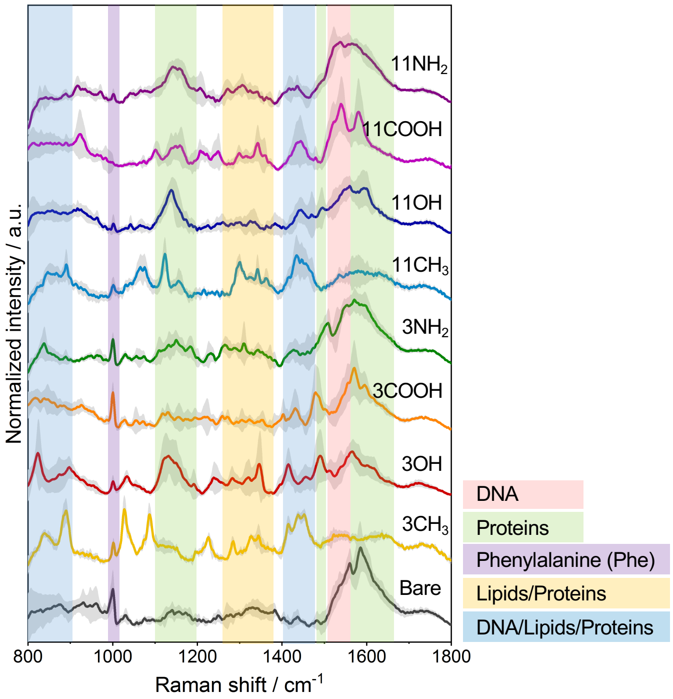

Supplementary Figure 8. Series of SERS spectra from purified extracellular vesicles (EVs) from MDA-MB-231 human breast cancer cells obtained from functionalized Au-nanopillars with various SAM-forming molecules. Each monolayer is referred to as: non-functionalized (bare), 1-propanethiol (3CH3), 3-mercapto-1-propanol (3OH), 3-mercaptopropionic acid (3COOH), 3-amino-1-propanethiol (3NH2), 1-undecanethiol (11CH3), 11-mercapto-1-undecanol (11OH), 11-mercaptoundecanoic acid (11COOH), 11-amino-1-undecanethiol (11NH2).

Supplementary Table 7. Discrimination of Hs578Bst (non-cancerous) and Hs578T (cancerous) cell lysates using PCA–LDA. The first PCs were calculated for each SAM-functionalization. Sensitivity, specificity and accuracy were calculated for cancerous cell lysates with respect to normal cell lysates from 9 individual PC1 (left). Each monolayer is referred to as: non-functionalized (bare), 1-propanethiol (3CH3), 3-mercapto-1-propanol (3OH), 3-mercaptopropionic acid (3COOH), 3-amino-1-propanethiol (3NH2), 1-undecanethiol (11CH3), 11-mercapto-1-undecanol (11OH), 11-mercaptoundecanoic acid (11COOH), 11-amino-1-undecanethiol (11NH2).

SAM molecule	Principal Component	Sensitivity (%)	Specificity (%)	Accuracy (%)
Bare	PC1	83.3	66.7	75.0
3CH3	PC1	50.0	100.0	75.0
3OH	PC1	66.7	50.0	58.3
3COOH	PC1	83.3	83.3	83.3
3NH2	PC1	83.3	100.0	91.7
11CH3	PC1	83.3	83.3	83.3
11OH	PC1	50.0	66.7	58.3
11COOH	PC1	50.0	33.3	41.7
11NH2	PC1	83.3	50.0	66.7

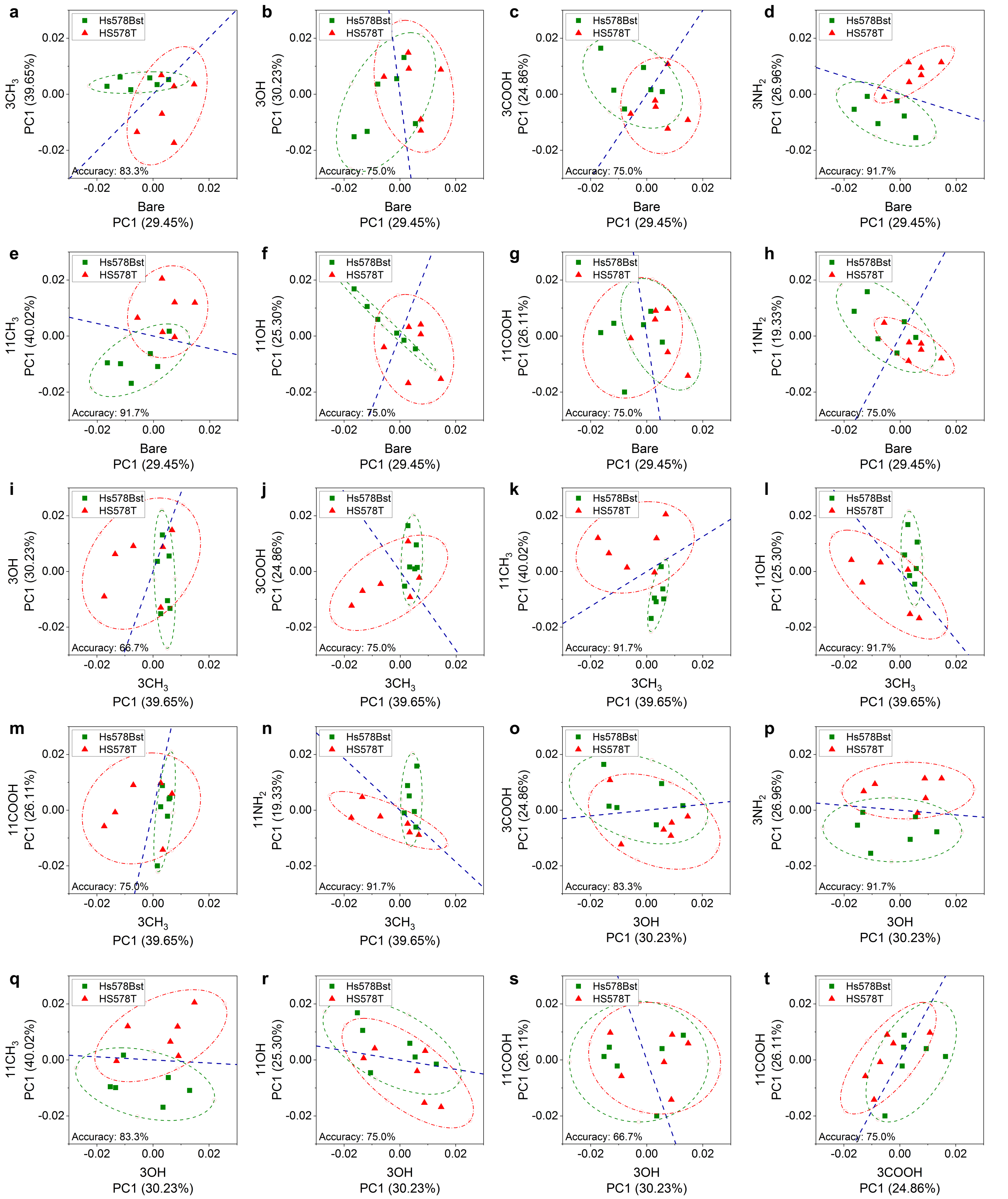

Supplementary Figure 9. Two-dimensional PCA scatter plots for Hs578Bst (non-cancerous) and Hs578T (cancerous) cell lysates using combinations of SAM-functionalization. (a–t) Each SAM is referred to as: non-functionalized (bare), 1-propanethiol ($3CH_3$), 3-mercapto-1-propanol (3OH), 3-mercaptopropionic acid (3COOH), 3-amino-1-propanethiol ($3NH_2$), 1-undecanethiol ($11CH_3$), 11-mercapto-1-undecanol (11OH), 11-mercaptoundecanoic acid (11COOH), 11-amino-1-undecanethiol ($11NH_2$). Blue dotted line was derived by LDA as a classification algorithm to separate the two groups. Red and green dotted boundaries represent confidence

intervals of the ± 1 s.d. of each group. Inset is the calculated accuracy in cancerous cell lysates (Hs578T) discrimination for each model. Additional combinations (u to ag) are included on the following page.

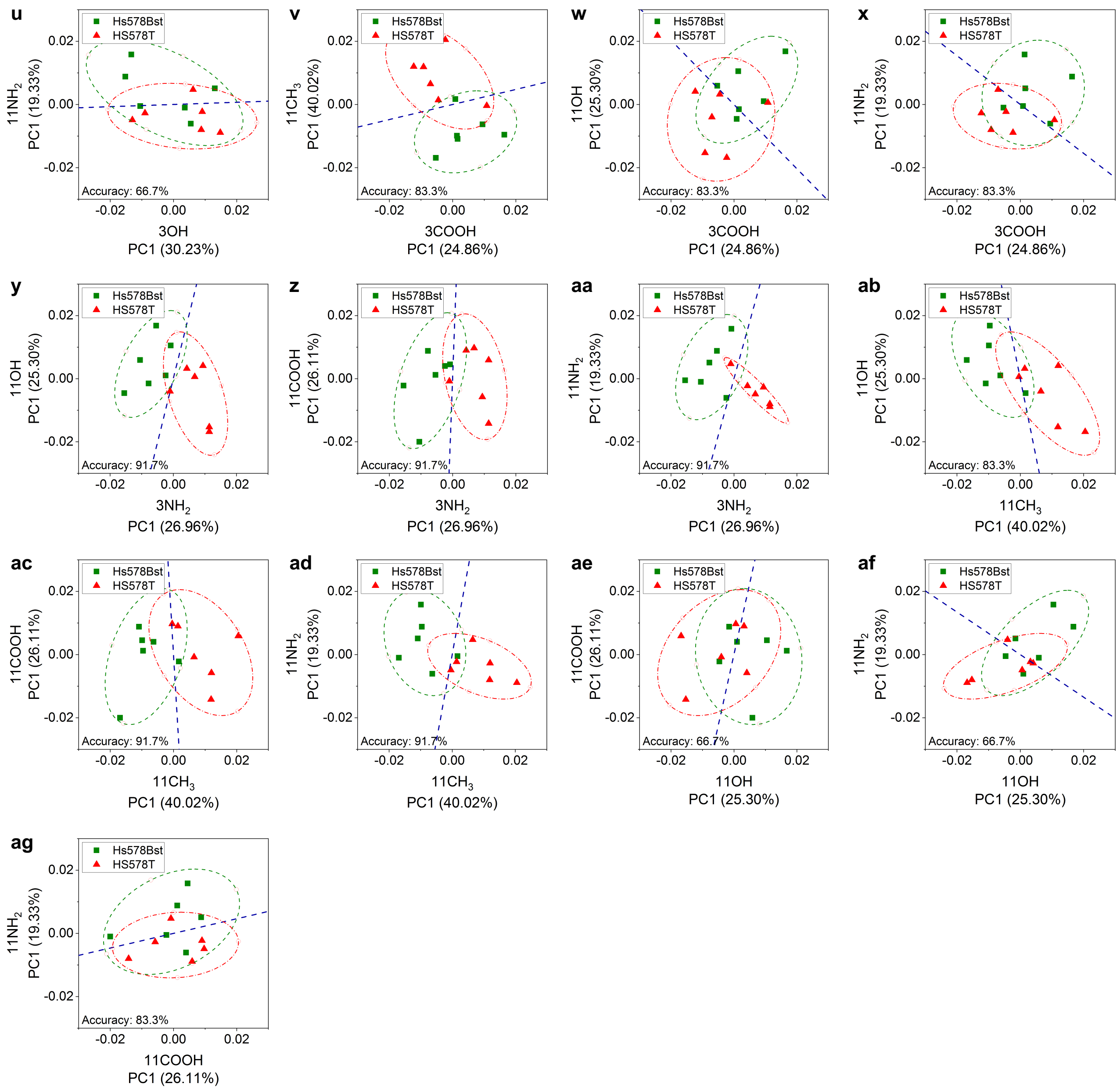

Supplementary Figure 9. (Continuation from previous page) Two-dimensional PCA scatter plots for Hs578Bst (non-cancerous) and Hs578T (cancerous) cell lysates using combinations of SAM-functionalization. (u–ag) Each SAM is referred to as: non-functionalized (bare), 1-propanethiol (3CH3), 3-mercapto-1-propanol (3OH), 3-mercaptopropionic acid (3COOH), 3-amino-1-propanethiol (3NH2), 1-undecanethiol (11CH3), 11-mercapto-1-undecanol (11OH), 11-mercaptoundecanoic acid (11COOH), 11-amino-1-undecanethiol (11NH2). Blue dotted line was derived by LDA as a classification algorithm to separate the two groups. Red and green dotted boundaries represent confidence intervals of the ± 1 s.d. of each group. Inset is the calculated accuracy in cancerous cell lysates (Hs578T) discrimination for each model.

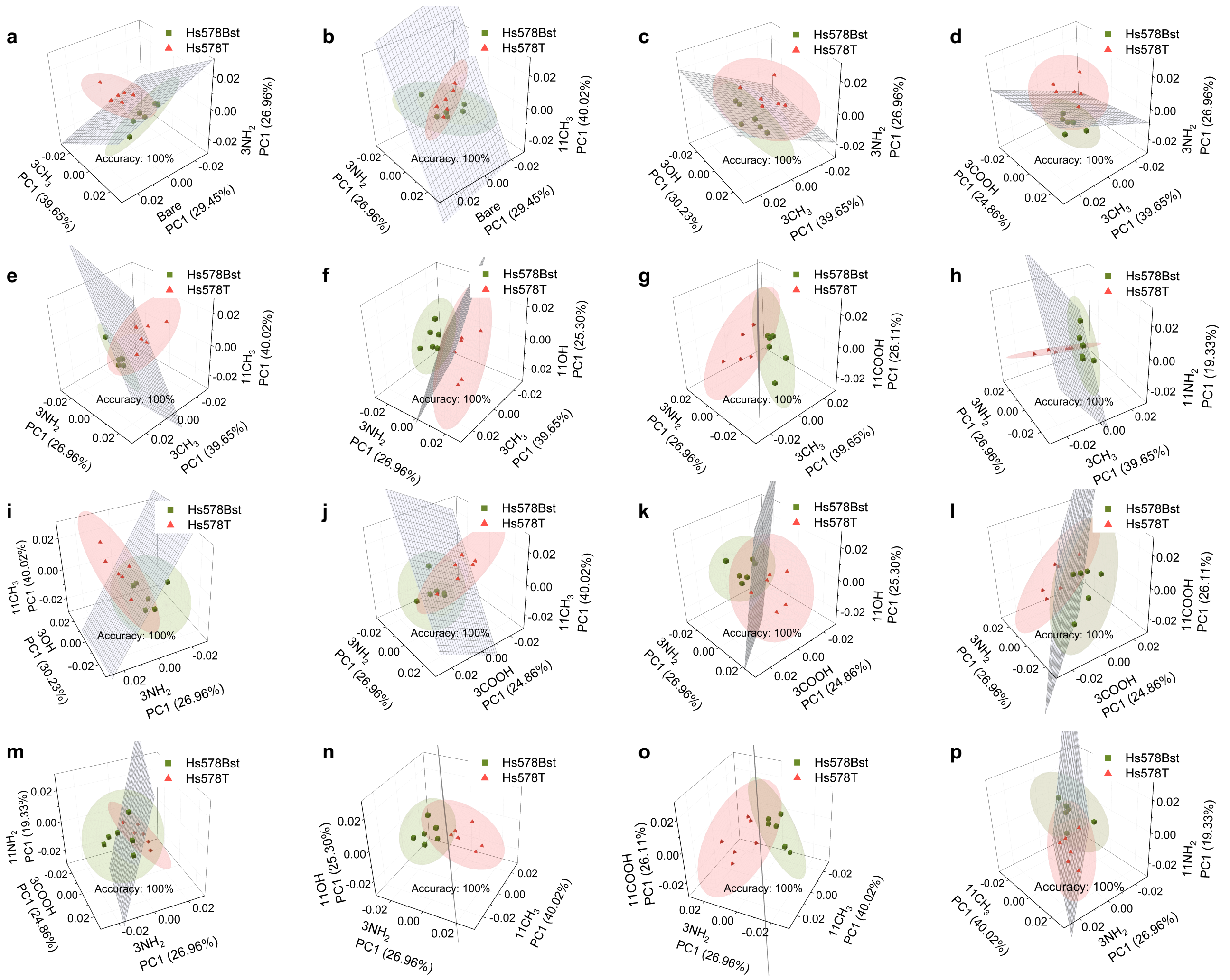

Supplementary Figure 10. Three-dimensional PCA scatter plots for Hs578Bst (non-cancerous) and Hs578T (cancerous) cell lysates using multiple SAM-functionalization. (a–p) Representative 3D scatter plots from combinations of different SAM-functionalizations that represent 100% accuracy in cancerous cell lysates discrimination. Each SAM is referred to as: non-functionalized (bare), 1-propanethiol ($3CH_3$), 3-mercapto-1-propanol (3OH), 3-mercaptopropionic acid (3COOH), 3-amino-1-propanethiol ($3NH_2$), 1-undecanethiol ($11CH_3$), 11-mercapto-1-undecanol (11OH), 11-mercaptoundecanoic acid (11COOH), 11-amino-1-undecanethiol ($11NH_2$). Blue planes depicting classification were derived from an LDA algorithm that separates the two groups. Red and green ellipsoids represent ± 1 s.d. of each group

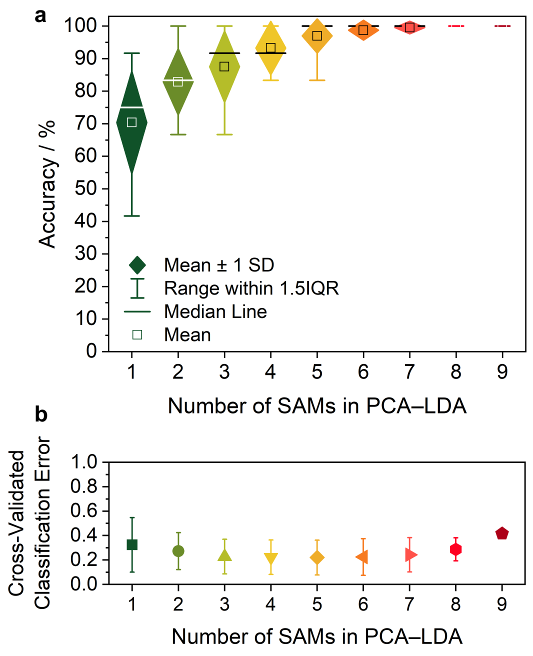

Supplementary Figure 11. Artificial-nose-empowered statistical multivariate analysis of model biological systems. (a) Mean accuracy for discrimination of cancerous cell lysates (Hs578T) from non-cancerous cell lysates (Hs578Bst) using PCA–LDA models, calculated at each dimensionality from all possible cross-combinations of the 9 PC1s up to 9 dimensions. (b) Evaluation of predictive performance of the PCA–LDA models in Figure 11a using leave-one-out cross-validation (LOOCV). CV-classification errors represent the misclassified fraction of the observations for each LOOCV model. Data represent mean ± 1 s.d. of all the models at each dimensionality. The mean accuracy of all possible PCA–LDA models increase with the addition of data dimensionality, reaching 100% accuracy when information from all the 9 sensors are combined. The CV models indicate that combining more than 7 can increase predictive inaccuracy of the system however with a potential problem of overfitting as the model complexity increases.

Supplementary References

1. Huang, Z., Fang, H. & Zhu, J. Fabrication of silicon nanowire arrays with controlled diameter, length, and density. *Adv. Mater.* **19**, 744–748 (2007).

2. Cheung, C. L., Nikolić, R. J., Reinhardt, C. E. & Wang, T. F. Fabrication of nanopillars by nanosphere lithography. *Nanotechnology* **17**, 1339–1343 (2006).

3. Kiraly, B., Yang, S. & Huang, T. J. Multifunctional porous silicon nanopillar arrays: antireflection, superhydrophobicity, photoluminescence, and surface-enhanced Raman scattering. *Nanotechnology* **24**, 245704 (2013).

4. Snow, A. W., Jernigan, G. G. & Ancona, M. G. Packing density of $HS(CH_2)_nCOOH$ self-assembled monolayers. *Analyst* **136**, 4935–4949 (2011).

5. Martines, E., Seunarine, K., Morgan, H., Gadegaard, N., Wilkinson C. D. W. & Riehle M. O. Superhydrophobicity and superhydrophilicity of regular nanopatterns. *Nano Lett.* **5**, 2097–2103 (2005).

6. Patankar, N. A. On the modeling of hydrophobic contact angles on rough surfaces. *Langmuir* **19**, 1249–1253 (2003).

7. Kim, D., Pugno, N. M. & Ryu, S. Wetting theory for small droplets on textured solid surfaces. *Sci. Rep.* **6**, 37813 (2016).

8. Sun. F. *et al*. Hierarchical zwitterionic modification of a SERS substrate enables real-time drug monitoring in blood plasma. *Nat. Commun.* **7**, 13437 (2016).

9. Bryant, M. A. & Pemberton, J. E. Surface Raman scattering of self-assembled monolayers formed from 1-alkanethiols: behavior of films at gold and comparison to films at silver. *J. Am. Chem. Soc.* **113**, 8284–8293 (1991).

10. Kudelski, A. Structures of monolayers formed from different HS—(CH2)2—X thiols on gold, silver and copper: comparitive studies by surface-enhanced Raman scattering. *J. Raman Spectrosc.* **34**, 853–862 (2003).

11. Bryant, M. A. & Pemberton, J. E. Surface Raman scattering of self-assembled monolayers formed from 1-alkanethiols at silver [electrodes]. *J. Am. Chem. Soc.* **113**, 3629–3637 (1991).

12. Sandhyarani, N. & Pradeep, T. Characteristics of alkanethiol self assembled monolayers prepared on sputtered gold films: a surface enhanced Raman spectroscopic investigation. *Vacuum* **49**, 279–284 (1998).

13. Michota, A., Kudelski, A. & Bukowska, J. Chemisorption of cysteamine on silver studied by surface-enhanced Raman scattering. *Langmuir* **16**, 10236–10242 (2000).

14. Michota, A., Kudelski, A. & Bukowska, J. Influence of electrolytes on the structure of cysteamine monolayer on silver studied by surface-enhanced Raman scattering. *J. Raman Spectrosc.* **32**, 345–350 (2001).

15. Michota, A., Kudelski, A. & Bukowska, J. Molecular structure of cysteamine monolayers on silver and gold substrates: comparative studies by surface-enhanced Raman scattering. *Surf. Sci.* **502–503**, 214–218 (2002).

16. Kudelski, A. Raman study on the structure of 3-mercaptopropionic acid monolayers on silver. *Surf. Sci.* **502–503**, 219–223 (2002).

17. Królikowska, A., Kudelski, A., Michota, A. & Bukowska, J. SERS studies on the structure of thioglycolic acid monolayers on silver and gold. *Surf. Sci.* **532–535**, 227-232 (2003).

18. Castner, D. G., Hinds, K. & Grainger, D. W. X-ray Photoelectron spectroscopy sulfur 2p study of organic thiol and disulfide binding interactions with gold surfaces. *Langmuir* **12**, 5083–5086 (1996).

19. Baio, J. E., Weidner, T., Brison, J., Graham, D. J., Gamble, L. J. & Castner, D. G. Amine terminated SAMs: Investigating why oxygen is present in these films. *J. Electron Spectrosc.* **172**, 2–8 (2009).

20. Yang, Z., Gonzalez-Cortes, A., Jourquin, G., Viré, J-C., Kauffmann, J-M. & Delplancke, J-L. Analytical application of self assembled monolayers on gold electrodes: critical importance of surface pretreatment. *Biosens. Bioelectron.* **10**, 789–795 (1995).

21. Bain, C. D., Troughton, E. B., Tao, Y. T., Evall, J., Whitesides, G. M. & Nuzzo, R. G. Formation of monolayer films by the spontaneous assembly of organic thiols from solution onto gold. *J. Am. Chem. Soc.* **111**, 321–335 (1989).

22. de Carvalho, D. F. *et al*. Surface-enhanced Raman scattering study of the redox adsorption of p-phenylenediamine on gold or copper surfaces. *Spectrochim. Acta A* **103**, 108–113 (2013).

23. Lin-Vien, D., Colthup, N. B., Fateley, W. G. & Grasselli, J. G. Aromatic and Heteroaromatic Rings. in *The Handbook of Infrared and Raman Characteristic Frequencies of Organic Molecules*, 277–306 (Academic Press Cambridge, 1991).

24. Hildebrandt, P. & Stockburger, M. Surface-enhanced resonance Raman spectroscopy of rhodamine 6G adsorbed on colloidal silver. *J. Phys. Chem.* **88**, 5935–5944 (1984).

25. Michaels, A. M., Nirmal, M. & Brus, L. E. Surface enhanced Raman spectroscopy of individual rhodamine 6G molecules on large Ag nanocrystals. *J. Am. Chem. Soc.* **121**, 9932–9939 (1999).

26. Kahraman, M., Daggumati, P., Kurtulus, O., Seker, E. & Wachsmann-Hogiu, S. Fabrication and characterization of flexible and tunable plasmonic nanostructures. *Sci. Rep.* **3**, 3396 (2013).

27. Watanabe, H., Hayazawa, N., Inouye, Y. & Kawata, S. DFT vibrational calculations of rhodamine 6G adsorbed on silver: analysis of tip-enhanced Raman spectroscopy. *J. Phys. Chem. B* **109**, 5012–5020 (2005).

28. Jensen, L. & Schatz, G. C. Resonance Raman scattering of rhodamine 6G as calculated using time-dependent density functional theory. *J. Phys. Chem. A* **110**, 5973–5977 (2006).

29. Kokaislová, A. & Matějka, P. Surface-enhanced vibrational spectroscopy of B vitamins: what is the effect of SERS-active metals used? *Anal. Bioanal. Chem.* **403**, 985–993 (2012).

30. Ren, W., Fang, Y. & Wang, E. A binary functional substrate for enrichment and ultrasensitive SERS spectroscopic detection of folic acid using graphene oxide/Ag nanoparticle hybrids. *ACS Nano* **5**, 6425–6433 (2011).

31. Kokaislová, A., Helešicová, T., Ončák, M. & Matějka, P. Spectroscopic studies of folic acid adsorbed on various metal substrates: does the type of substrate play an essential role in temperature dependence of spectral features? *J. Raman Spectrosc.* **45**, 750–757 (2014).

32. Love, J. C., Estroff, L. A., Kriebel, J. K., Nuzzo, R. G. & Whitesides, G. M. Self-assembled monolayers of thiolates on metals as a form of nanotechnology. *Chem. Rev.* **105**, 1103–1170 (2005).

33. Zhao, J., Luo, L., Yang, X., Wang, E. & Dong, S. Determination of surface pKa of SAM using an electrochemical titration method. *Electroanalysis* **11**, 1108–1113 (1999).

34. Fears, K. P., Creager, S. E. & Latour, R. A. Determination of the surface pK of carboxylic- and amine-terminated alkanethiols using surface plasmon resonance spectroscopy. *Langmuir* **24**, 837–843 (2008).

35. Munakata, H., Oyamatsu, D. & Kuwabata, S. Effects of omega-functional groups on pH-dependent reductive desorption of alkanethiol self-assembled monolayers. *Langmuir* **20**, 10123–10128 (2004).

36. Hess, B., Kutzner, C., van der Spoel, D. & Lindahl, E. GROMACS 4: Algorithms for highly efficient, load-balanced, and scalable molecular simulation. *J. Chem. Theory Comput.* **4**, 435–447 (2008).

37. Wright, L. B., Rodger, P. M., Corni, S. & Walsh, T. R. GolP-CHARMM: First-principles based force fields for the interaction of proteins with Au(111) and Au(100). *J. Chem. Theory Comput.* **9**, 1616–1630 (2013).

38. Jorgensen, W. L., Chandrasekhar, J., Madura, J. D., Impey, R. W. & Klein, M. L. Comparison of simple potential functions for simulating liquid water. *J. Chem. Phys.* **79**, 926–935 (1983).

39. Reimers, J. R., Ford, M. J., Marcuccio, S. M., Ulstrup, J. & Hush, N. S. Competition of van der Waals and chemical forces on gold–sulfur surfaces and nanoparticles. *Nat. Rev. Chem* **1**, 0017 (2017).

40. Tachibana, M., Yoshizawa, K., Ogawa, A., Fujimoto, H. & Hoffmann, R. Sulfur−gold orbital interactions which determine the structure of alkanethiolate/Au(111) self-assembled monolayer systems. *J. Phys. Chem. B* **106**, 12727–12736 (2002).

41. Kondoh, H. *et al*. Adsorption of thiolates to singly coordinated sites on Au(111) evidenced by photoelectron diffraction. *Phys. Rev. Lett.* **90**, 066102 (2003).

42. Bürgi, T. Properties of the gold-sulphur interface: from self-assembled monolayers to clusters. *Nanoscale* **7**, 15553–15567 (2015).

43. Vanommeslaeghe, K. *et al*. CHARMM general force field: A force field for drug-like molecules compatible with the CHARMM all-atom additive biological force fields. *J. Comput. Chem.* **31**, 671–690 (2010).

44. Nosé, S. A molecular dynamics method for simulations in the canonical ensemble. *Mol. Phys.* **52**, 255–268 (1984).

45. Hoover, W. G. Canonical dynamics: equilibrium phase-space distributions. *Phys. Rev. A* **31**, 1695–1697 (1985).

46. Hess, B., Bekker, H., Berendsen, H. J. C. & Fraaije, J. G. E. M. LINCS: A linear constraint solver for molecular simulations. *J. Comput. Chem.* **18**, 1463–1472 (1997).

47. Humphrey, W., Dalke, A. & Schulten, K. VMD: Visual molecular dynamics. *J. Mol. Graph.* **14**, 33–38 (1996).
